# Acupuncture in remodeling the tumor microenvironment: current status and challenges

**DOI:** 10.3389/fimmu.2026.1737746

**Published:** 2026-04-01

**Authors:** Jinming Liu, Yanjun Jin, Yan Ou, Linbo Liu, Shanqi Guo, Binxu Sun

**Affiliations:** 1 First Teaching Hospital of Tianjin University of Traditional Chinese Medicine, Tianjin, China; 2 National Clinical Research Center for Chinese Medicine, Tianjin, China; 3 School of Chinese Medicine, Tianjin University of Traditional Chinese Medicine, Tianjin, China

**Keywords:** acupuncture, breast cancer, clinical symptoms, mechanism, review, tumor microenvironment

## Abstract

The tumor microenvironment (TME) is a dynamic ecosystem in which malignant, immune, stromal, and vascular compartments continuously interact, and plays a key role in tumor initiation, development and treatment resistance. In recent years, acupuncture, as an ancient neuromodulatory intervention means of traditional medicine, has shown promise in supportive oncology by attenuating chemotherapy- and radiotherapy-induced toxicities, modulating immunity, and improving quality-of-life metrics. Yet, a mechanistic framework that links acupuncture to TME reprogramming remains to be established. From the perspective of TME, we reviews the latest research status of acupuncture anti-tumor mechanism. Evidence synthesized indicates that acupuncture (i) triggers apoptosis of malignant cells, (ii) re-educates innate (NK, macrophage, dendritic, and mast) and adaptive (T and B lymphocyte) immune subsets, and (iii) normalizes tumor vasculature, so as to inhibit tumor growth and metastasis, synergize chemotherapy and immunotherapy, and promote physical rehabilitation. We further outline opportunities and challenges for translating acupuncture into evidence-based oncology. Using breast cancer as a paradigm, we emphasize the need to evaluate the role of acupuncture in different molecular subtypes and within integrative survivorship care. Furthermore, we aim to link its benefit of relieving symptoms with TME modulation mechanisms, thereby constructing an integrated evidence chain connecting “clinical symptoms—acupuncture intervention—TME modulation—long-term prognosis.”. Interdisciplinary trials that couple mechanistic TME readouts with robust clinical endpoints are now warranted to definitively establish the efficacy and safety of acupuncture in cancer care.

## 1 Introduction

Cancer remains the second leading cause of death globally. Morbidity and mortality are still increasing worldwide. In 2025, 2.04 million new cases and 618120 deaths occurred in the U.S. cancer statistics project (1). These numbers translate into unsustainable healthcare costs and identify urgent public health priorities.

Multimodal regimens have replaced monotherapy. Surgery, radiotherapy, and systemic agents are now combined patient-by-patient, doubling 5-year survival relative to single-modality eras (2). Yet, the heterogeneity among patients precludes true precision medicine (3). Acquired resistance and off-target toxicities further limit survival gains (4, 5). Consequently, dissecting clonal evolution and microenvironmental crosstalk are prerequisites for next-generation interventions.

The tumor microenvironment (TME) is an integral component of tumors and is widely recognized for its dynamic role in regulating cancer progression and influencing treatment efficacy (6). It integrates immune infiltration, cytokine gradients, extracellular matrix, and aberrant vasculature. Together, they promote chronic inflammation, immune evasion, and neovascularization (7). By cultivating resistance and exacerbating off target toxicity, TME reduces cytotoxicity and the efficacy of targeted drugs resistance and exacerbating off-target toxicity, the TME curtails the efficacy of cytotoxic and targeted agents (8). Therefore, the focus of treatment has shifted from tumor centered to TME centered strategies.

**Figure 1:**
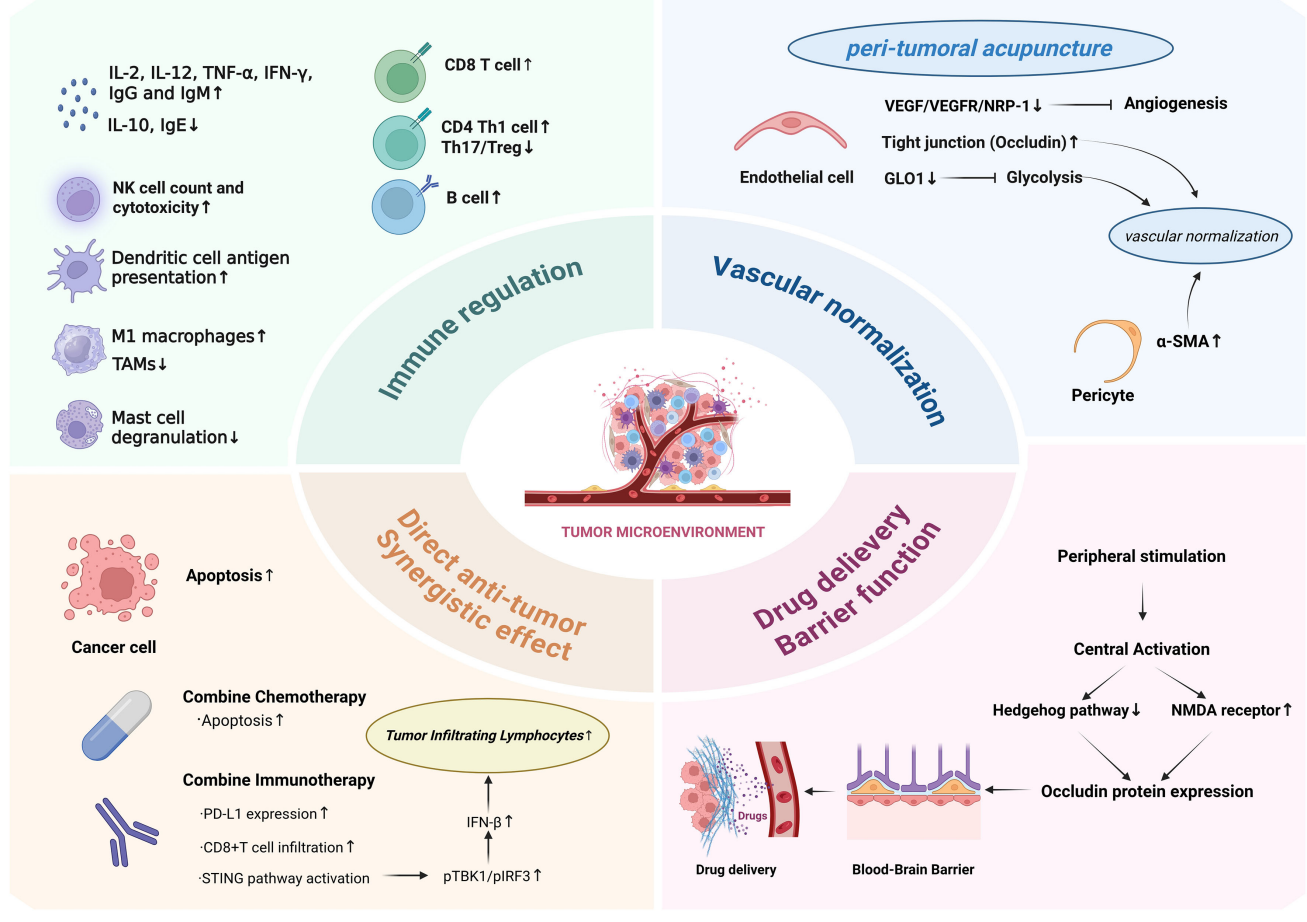
The intervention effect of acupuncture on tumor microenvironment. Distal acupuncture can directly induce apoptosis in tumor cells and enhance tumor suppression when combined with chemotherapy or immunotherapy. It improves the tumor immune microenvironment and activates antitumor immune responses by modulating the infiltration and function of innate immune cells (such as NK cells, macrophages, and dendritic cells) and adaptive immune cells (T and B lymphocytes), as well as by balancing cytokine levels. Additionally, acupuncture upregulates NMDA receptors in the brain central nervous system, influences the Hedgehog signaling pathway, and further downregulates Occludin protein expression, thereby increasing blood-brain barrier permeability and enhancing drug delivery efficiency at the tumor site. Notably, peritumoral acupuncture can downregulate the VEGF/VEGFR pathway and targets such as GLO1, promoting tight junctions in tumor vascular endothelial cells and pericyte coverage, leading to vascular normalization. A image created with BioRender.com.

Acupuncture, a minimally invasive neuromodulatory stimulus, evokes systemic reflexes. It has been reported to ameliorate infection, allergy, autoimmunity, and immunodeficiency (9). Favorable safety profiles underlie its increasing clinical adoption (10). In oncology, acupuncture augments anti-tumor immunity, mitigates treatment-related toxicities, and improves quality-of-life metrics (11). Mechanistically, acupuncture triggers apoptosis of malignant cells and potentiates cytotoxic drugs (12). It further accelerates postoperative recovery through neuroendocrine regulation (13). Moreover, acupuncture reprograms intratumoral immune cells to restrain progression and metastasis (14). Collectively, acupuncture is becoming a targeted treatment modality for TME. Analyzing its multimodal impact on TME will provide information for evidence-based implementation. Here, we systematically synthesize recent advances in the mechanisms of acupuncture-mediated TME reprogramming and, using breast cancer as a case study, emphasize the importance of linking clinical symptom management with TME mechanisms. Converging data show that acupuncture restrains tumors by reprogramming TME cellularity and function, positioning it as a complementary therapeutic strategy. (see **Figure 1**)

## 2 Direct antitumor effects and combination therapy of acupuncture

Five decades of research have condensed cancer biology into eight hallmarks (15). These hallmarks include sustained proliferation, evasion of growth suppressors, apoptosis resistance, limitless replication, angiogenesis, and metastasis. Aberrant proliferation is the universal driver of malignancy. Resulting masses compress adjacent tissue, invade locally and seed distant organs. Tumor size predicts prognosis; invasion and metastasis dictate mortality (16, 17). Inducing cancer cell death remains the primary therapeutic goal. When conventional therapies fail, non-pharmacological options such as acupuncture are explored. Acupuncture reduces tumor volume by up to 45% in rat Walker-256 hepatoma, gastric and subcutaneous models (18). Osteosarcoma-bearing mice exhibit 30% slower growth when ≥3 acupoints are stimulated. These changes coincide with reduced densities of tumor vessels, lymphatics and nerves, and a 60% drop in pulmonary metastases (19, 20). Timing determines efficacy. Day-3 acupuncture suppresses tumor burden by 40%, whereas day-7 treatment doubles tumor volume (20). The therapeutic window is therefore confined to early-stage disease. Late-stage tumor promotion has not previously been reported. Prospective mapping of the time-dependency is now required.

Monotherapy inevitably selects for resistance. Rational combination strategies are therefore essential. First-line chemotherapy still depends on apoptosis induction (21). Electroacupuncture (EA) enhances cisplatin-mediated tumor suppression in patients with stage IIb–IIIb cervical squamous cell carcinoma, yielding greater tumor shrinkage than chemotherapy alone (22). The synergy stems from increased apoptosis. EA-capeOX doubles apoptotic index and maximally reduces tumor volume by day 7 (23). Beyond chemosensitization, acupuncture improves quality-of-life metrics and potentially extends survival (24). Evidence remains fragmented and tumor-centric. Robust trials and mechanistic insights are lacking. Multicenter, randomized, mechanism-anchored trials are now warranted.

## 3 Acupuncture and the tumor immune microenvironment

The tumor immune microenvironment (TIME) encompasses all immune constituents within the TME. These constituents interact to drive or suppress anti-tumor immunity (25). Immune cells dictate TIME status. Tumors are classified as ‘cold’ or ‘hot’ according to immune infiltration patterns (26). Cold tumors lack CTLs and evade immune surveillance. Hot tumors harbor activated CTLs, NK cells and DCs that mount effective anti-tumor responses. Immune cell density and diversity predict treatment response and survival (27, 28). Converting cold into hot tumors is now a research priority. Acupuncture remodels the TIME by boosting immunity and restoring homeostasis (29). This translates into improved outcomes and quality of life (14).

### 3.1 Adaptive immunity

The adaptive immune system eliminates specific antigens through clonally restricted receptors and effector molecules. Essential for anti-pathogen and anti-tumor immunity, it drives autoimmunity when dysregulated (30). T and B lymphocytes execute antigen-specific responses. In the TIME, inhibitory cytokines, exhausted lymphocytes and suppressive infiltrates paralyze adaptive immunity (31).

#### 3.1.1 T lymphocytes

CD8^+^, CD4^+^ and Treg subsets dominate cancer immunotherapy studies. CD8^+^ T cells kill tumor cells via perforin/granzyme or FAS-FASL ligation. Intratumoral CD8^+^ density predicts survival. Pretreatment osteosarcoma biopsies (n=124) show that abundant CD8^+^ T cells correlate with longer overall survival (32). Merkel-cell carcinoma displays the same association (33). Chronic antigen exposure and epigenetic rewiring exhaust CD8^+^ T cells and impair tumor control (34). Hence, checkpoint monotherapy frequently fails (35). CD4^+^ T cells exert context-dependent pro- or anti-tumor activity (36). Th1 cells license CD8^+^ activity and secrete IL-2, TNF-α and IFN-γ. Th2 and Th17 cytokines instead fuel tumor growth. Tregs curb autoimmunity but also suppress anti-tumor immunit (37). In cancer, however, Tregs inhibit effector cell function, leading to immune evasion. Acupuncture expands and activates CD3^+^CD8^+^ pools while rebalancing CD4^+^ subsets.

EA increases splenic CD3^+^CD4^+^ and CD3^+^CD8^+^ T-cell subsets and plasma IL-2 levels, albeit with weaker analgesia than morphine, in a rat model of breast-cancer bone pain (38). Acupuncture preserves CD8^+^ T-cell numbers and reverses drug-induced immunosuppression in patients with metastatic bone pain (39). Acupuncture also preserves hematopoietic and immune function after chemotherapy. In 28 cancer patients, one month of acupuncture during chemotherapy maintained CD3^+^, CD4^+^, CD8^+^ counts and the CD4^+^/CD8^+^ ratio (40). EA alleviates chemotherapy-induced myelosuppression and reshapes TME immune infiltration and cytokine profiles in NSCLC mice (41, 42). EA expands peripheral hematopoietic stem and leukocyte subsets, indicating bone-marrow niche reconstruction. Intratumorally, EA increases CD8^+^ infiltration and IL-2/IFN-γ while curbing Th17 and Treg fractions. EA boosts bone-marrow sympathetic density and PACAP–PAC1 signaling, coupling hematopoietic protection to anti-tumor immunity (41). Cancer−related fatigue (CRF) affects >90% of patients during chemotherapy (43). IL-6 and IL-1-mediated neuroinflammation precipitate fatigue (44, 45). Leptin, a key hormone in energy metabolism, is concurrently elevated (46). Acupuncture suppresses leptin–AMPK signaling, rescues neuronal mitochondria and restores CD3^+^, CD4^+^ T cells and immunoglobulin levels, reversing fatigue (45).

Acupuncture also sensitizes tumors to immunotherapy. EA downregulates HDAC1, recruits CD8^+^ T cells and curbs triple-negative breast cancer growth (47). EA plus HDAC inhibitor achieves 60% tumor control, confirming TIME remodeling (48). MSS colorectal cancer, an immune-desert subtype, resists checkpoint blockade (49). EA plus anti–PD-1 converts MSS tumors from cold to hot (50). EA upregulates PD-L1, recruits CD8^+^ T cells and engages STING. Optimal immunomodulation occurs at 1.0 mA, coinciding with dampened neuronal firing. Thus, EA operates via the neuro-endocrine-immune axis (10, 51). Translation to randomized trials is now justified. Acupuncture enhances CD3^+^, CD4^+^ and CD8^+^ T-cell function, potentiates immunotherapy and depletes Th17 and Treg cells.

#### 3.1.2 B lymphocytes

B cells underpin humoral immunity. They secrete antibodies that bind mutation-derived or post-translationally modified neoantigens. Activated B cells further license T cells via antigen presentation and co-stimulation (52). TME-derived signals impair B-cell proliferation, differentiation, antibody output and complement activation, enabling escape. Acupuncture rescues B-cell counts and rebalances immunoglobulin titers. It reverses chemotherapy- or analgesia-driven B-cell loss (39, 53). Antibody output is bidirectionally tuned by acupuncture. In autoimmunity (rheumatoid arthritis, obesity), acupuncture lowers IgG, IgA and IgM (54, 55). Conversely, in cancer-related immunosuppression, it raises IgG and IgM (56).

### 3.2 Innate immunity

Innate immunity mounts rapid, non-specific defenses that precede adaptive responses (57). These comprise physical barriers, effector cells and molecules that block pathogens and initiate downstream inflammation and adaptive immunity (58). NK cells, macrophages and dendritic cells clear pathogens, regulate inflammation and defend the host. Within the TIME, these cells suppress tumors via direct cytotoxicity or by priming adaptive immunity (59). Acupuncture boosts innate-cell numbers and activity, reinforcing anti-tumor defense.

#### 3.2.1 Natural killer cells

Natural killer (NK) cells are innate cytotoxic lymphocytes that act as a first-line defense against neoplastic, senescent or infected cells. They eliminate target cells without prior sensitization, as they do not require antigen presentation (60). Cytotoxicity is mediated by perforin–granzyme exocytosis or by engagement of death receptors belonging to the tumor-necrosis factor (TNF) superfamily (61). Tumors progressively evade NK-cell surveillance by up-regulating inhibitory ligands and recruiting immunosuppressive myeloid and regulatory T cells (62). The effect of acupuncture on NK-cell number and function has been investigated in both clinical and pre-clinical settings. In patients with anxiety disorders, acupuncture restored NK-cell activity, which remained elevated for at least four weeks after the final session. In women with anxiety, NK cell activity remained elevated for one month following acupuncture treatment (63). EA attenuated surgery-induced immunosuppression in craniotomy patients, as evidenced by increased NK-cell counts and serum IgM/IgA (64). Collectively, these data indicate that acupuncture can re-establish NK-cell homeostasis in immunocompromised hosts.

It has been hypothesized that acupuncture augments anti-tumor immunity, at least in part, by activating NK cells (65). In post-operative colorectal-cancer patients, acupuncture increased the peripheral NK-cell fraction and was associated with improved gastrointestinal and immune recovery (66). Transcutaneous electrical acupoint stimulation restored NK-cell numbers in patients with non-small-cell lung cancer during the perioperative phase, and concurrently reduced plasma TNF-α and IL-6, stabilized hemodynamics, lowered anesthetic requirement and shortened hospitalisation (67). In chemotherapy-treated patients, acupuncture mitigated treatment-related immune suppression and preserved NK-cell cytolytic activity (53). In cyclophosphamide-treated mice, EA enhanced NK-cell cytotoxicity and increased systemic IL-2, IL-12, TNF-α and IFN-γ, an effect that was dependent on DREAM–NF-κB signaling (68). When EA was combined with chemotherapy in cervical-cancer patients, tumor control was improved and corresponded with expanded NK-cell pools and reduced lesion volume (22).

Pre-clinical data show that acupuncture modulates splenic and peripheral NK-cell compartments by releasing β-endorphin, dampening hypothalamic–pituitary–adrenal (HPA) axis output and activating vagal afferents (69). β-Endorphin, an endogenous opioid released during HPA-axis activation, reaches immune cells through the systemic circulation (70). Acupuncture-induced β-endorphin up-regulates perforin, granzymes and TNF-family ligands, and amplifies IFN-γ secretion by NK cells (71, 72). EA also activates choline-acetyltransferase-positive (ChAT^+^) neurons in the dorsal motor nucleus of the vagus, thereby increasing the NK-cell fraction and cytolytic capacity while curbing accumulation of myeloid-derived suppressor cells (MDSCs), and consequently retarding 4T1-luc2 breast-tumor growth in mice (73). Together, these pathways converge to strengthen NK-cell-dependent anti-tumor immunity.

#### 3.2.2 Macrophages

Macrophages arise from bone-marrow hematopoietic stem cells and orchestrate phagocytosis, antigen presentation, inflammatory control and tissue homeostasis (74). In many solid tumors, macrophages account for up to 50% of the total cellularity (75). Macrophages polarize along a continuum, but are commonly classified into pro-inflammatory M1 or anti-inflammatory M2 extremes. Within the TIME, M1 macrophages restrain malignancy, whereas M2-polarised tumor-associated macrophages (TAMs) foster progression. M1 cells directly phagocytose malignant cells, trigger apoptosis and cross-talk with the adaptive arm by activating antigen-presenting cells and recruiting CD8^+^ T, Th1 and NK cells (76). Conversely, TAMs promote angiogenesis, suppress anti-tumor immunity, facilitate metastasis and blunt therapeutic efficacy (77). Therapeutic reprogramming of TAMs toward an M1-like state is therefore under intense investigation.

Acupuncture studies have interrogated macrophage abundance, polarization and secretory profiles. In a rat model of chronic obstructive pulmonary disease, acupuncture lowered macrophage counts in bronchoalveolar-lavage fluid, curtailed inflammatory infiltrates and improved lung mechanics (78). In rheumatoid-arthritis models, acupuncture mitigated inflammation and pain, effects linked to suppressed M1 polarization and reduced IL-1β (79). Peri-tumor electroacupuncture (EA) shifted TAM polarity toward an M1 phenotype, leading to decreased microvessel density, increased pericyte coverage and enhanced vascular maturation (80). This reprogramming was dependent on down-regulation of glyoxalase-1 (GLO1) and activation of the methylglyoxal–AGE/RAGE axis (81). These data provide the first demonstration that EA curtails angiogenesis by enforcing an M1-like TAM signature. The intersection of acupuncture and TAM biology remains exploratory and warrants mechanistic dissection.

#### 3.2.3 Dendritic cells

Dendritic cells (DCs) integrate innate and adaptive immunity by acquiring, processing and presenting antigens that initiate and sculpt T-cell responses (82). In cancer hosts, DCs relay tumor antigens to effector T cells and secrete cytokines that license anti-tumor CD4^+^ and CD8^+^ T-cell immunity (83). Yet, defective DC differentiation or trafficking frequently blunts tumor-specific T-cell priming. In 4T1 mammary-tumor-bearing mice, acupuncture plus anti-PD-1 therapy synergistically curtailed tumor growth. This efficacy coincided with expansion of CD5^+^ DCs, systemic accumulation of CD4^+^ and CD8^+^ T cells and heightened IL-2, IL-6, TNF-α and IFN-γ concentrations (84). CD5, a transmembrane glycoprotein mandatory for optimal effector T-cell activation, is selectively induced on DCs by IL-6 (85). Acupuncture also shapes the local DC pool at acupoint sites, probably via neurogenic inflammation within dermal tissue (86).

#### 3.2.4 Mast cells

Mast cells (MCs) are innate immune cells that reside in perivascular and perineural niches. They are best recognized for orchestrating allergic responses and anti-helminth immunity (87). Recent tumor-biology studies reveal that MC density and their inflammatory cargo within the tumor microenvironment (TME) exert context-dependent, dichotomous effects on gastrointestinal malignancies (88, 89). In pancreatic-cancer patients with visceral pain, peritumoral tissues exhibit elevated MC counts and high levels of histamine, tryptase and nerve growth factor (NGF), implicating MC degranulation in visceral hypersensitivity (90). MCs further amplify angiogenesis by releasing VEGF, FGF-2, PDGF and angiopoietin-1, thereby fueling tumor expansion (91). Acupuncture suppresses MC degranulation in both allergic and inflammatory settings. In a rat model of irritable-bowel syndrome, acupuncture curbed aberrant MC proliferation and activation in colonic mucosa, normalized substance P (SP) and vasoactive-intestinal-peptide (VIP) release and ameliorated visceral hypersensitivity (92). EA also attenuated MC infiltration and degranulation in atopic-dermatitis rats through cannabinoid CB2 receptors, concomitantly lowering IgE and other immune-active mediators (93). Acupuncture may further recruit endogenous opioid pathways to curb MC degranulation and relieve pain (94). These mechanisms collectively illustrate how acupuncture tunes MC-mediated immune responses.

## 4 Acupuncture and the vasculature in tumors

Angiogenesis is an obligate prerequisite for tumor expansion and metastatic spread (95). Initially, neoplastic cells rely on diffusion for nutrient uptake. Once the lesion exceeds ~2 mm, diffusion becomes insufficient and a dedicated vascular network must be forged to deliver oxygen and nutrients and to evacuate metabolic waste (96, 97). Tumor angiogenesis is orchestrated by a multicellular interplay among malignant cells, vascular cells and pro-angiogenic cues (98). Within the tumor microenvironment (TME), chronic overproduction of pro-angiogenic signals generates immature, chaotic and dysfunctional vascular beds. T These aberrant vessels are tortuous, hyperpermeable and inefficient, fostering hypoxia and lactate build-up (99). Sparse pericyte coverage and weakened endothelial–pericyte crosstalk further undermine vascular maturity and stability (100). Consequently, perfusion is impaired and drug delivery is compromised. Jain’s 2001 concept of vascular normalization posits that transient pharmacological repair, rather than destruction, of tumor vessels can curb metastasis and enhance drug delivery (101). Subsequent work indicates that acupuncture can reprogram vascular cells and angiogenic cues to normalize tumor vasculature.

### 4.1 Vascular endothelial cells

Endothelial cells (ECs) line the luminal surface of vessels and constitute the central cellular driver of angiogenesis. In healthy tissue, ECs are tightly juxtaposed, creating a selective vascular barrier. In tumors, ECs down-regulate adhesion molecules, adopt a loose configuration and thereby permit intravasation and metastatic dissemination (102). Paracellular leakage elevates interstitial fluid pressure, reducing perfusion, aggravating hypoxia and ultimately collapsing vessels (103). The resultant chaotic vasculature creates large, under-perfused and drug-inaccessible tumor regions (104). Emerging evidence shows that inhibition of glycolysis in tumor ECs can drive vascular normalisation (105, 106). As outlined above, endothelial GLO1 and the glycolytic methylglyoxal-detoxification axis are proximal targets of acupuncture (81). Acupuncture down-regulates GLO1, curbs angiogenesis and reinforces inter-endothelial junctions, peaking 72 h post-stimulus. Electroacupuncture-evoked decreases in glycolytic flux and methylglyoxal metabolites corroborate GLO1 as the proximal enzymatic target. Hypoxic tumor regions release VEGF, igniting pro-angiogenic signaling cascades (107). Acupuncture blunts tumor angiogenesis by down-regulating endothelial VEGF-A, VEGFR-2 and the co-receptor neuropilin-1 (NRP-1) (108). NRP-1, an obligate co-receptor for developmental vascular patterning, potentiates VEGFR signaling in ECs (109).

Beyond the tumor core, the blood–brain barrier (BBB) constitutes a formidable obstacle to brain-tumor therapy (110). Macromolecular therapeutics fail to achieve cerebrovascular penetration, and local delivery is constrained by surgical invasiveness and limited diffusion (111). The BBB is constituted by brain microvascular ECs joined by continuous tight junctions. Electroacupuncture delivered at 2/100 Hz, 3 mA, 6–6 s cycles for 40 min transiently opens the BBB, augments cerebral paclitaxel levels and potentiates anti-glioma activity (112, 113). The mechanism involves endothelial NMDA-receptor activation, Hedgehog-pathway modulation and occludin displacement at tight junctions. Whether BBB opening is acupoint-specific remains contentious. Some studies restrict efficacy to craniofacial acupoints (112), whereas others invoke a primitive vascular system (PVS) that links remote spinal or limb acupoints to the brain, bypassing the BBB (114).

### 4.2 Pericytes

Pericytes are mesenchymal mural cells that encase capillaries and crosstalk with ECs through juxtacrine and paracrine signals to sustain vascular maturation and stability (115). Within tumors, pericytes play paradoxical roles. They can impair drug delivery and immune access by increasing mural coverage of tumor vessels (116). Conversely, dense pericyte coverage can restrain tumor-cell dissemination (117). Peri-tumor acupuncture increases pericyte coverage and drives vascular normalization in superficial tumors. Acupuncture up-regulates α-SMA, expands differentiated functional vessels and leaves total vascular density unchanged (118). This normalization elevates intratumoral paclitaxel concentrations in murine breast-cancer models. Collectively, acupuncture functions as a biological navigation system that redistributes drugs within tumor tissue and amplifies therapeutic efficacy (119). Importantly, acupuncture outperforms anti-angiogenic agents in tightening endothelial junctions and expanding pericyte coverage (81), underscoring its adjunctive potential in combination regimens.

## 5 Acupuncture in breast cancer: TME mechanisms and symptom management

Breast cancer, the most prevalent malignancy in women worldwide, provides an ideal model for an “Acupuncture—TME Mechanism—Symptom Management” framework. This utility stems from its clearly defined molecular subtypes, distinctive TME features, diverse therapeutic options, and stage-dependent symptomatology.

### 5.1 Acupuncture mechanisms in different subtypes and symptom management

Based on HER2 and hormone receptor status, breast cancer is classified into three main subtypes: Luminal A/B, HER2-enriched, and triple-negative breast cancer (TNBC). This classification is clinically important because each subtype displays distinct tumor microenvironment (TME) heterogeneity, guiding different treatment approaches (120). Among these, TNBC—though aggressive and associated with poorer prognosis—often presents a more immunogenic TME, featuring higher levels of tumor-infiltrating lymphocytes, PD-L1 expression, and tumor mutational burden, making it more amenable to immunotherapy (121). Preclinical studies suggest that acupuncture may promote tumor cell apoptosis and enhance immune cell infiltration (including CD5+ dendritic cells and CD4+/CD8+ T lymphocytes) in TNBC models, potentially synergizing with immunotherapies (84). In contrast, Luminal and HER2-enriched subtypes typically exhibit an immunologically “cold” TME with lower immune infiltration, which may limit their response to immunotherapy and reduce any direct immunomodulatory effects of acupuncture. However, acupuncture has been shown to effectively relieve treatment-related side effects in these patients—such as hot flashes, pain, neuropathy, fatigue, and myelosuppression—through modulation of neuro-inflammatory and neuro-endocrine-immune pathways (24). For example, in ovariectomized rat models, electroacupuncture has been found to significantly upregulate hypothalamic aromatase expression and activity at both the mRNA and protein levels. This modulation subsequently influences GnRH neuronal function, leading to an improvement in vasomotor symptoms (122). Furthermore, electroacupuncture can reduce the release of key pro-inflammatory cytokines, such as TNF-α and IL-1β, in both the spinal cord and peripheral circulation (123). While current evidence clarifies how acupuncture alleviates symptoms, it remains unclear whether these benefits involve direct or indirect effects on the TME. Future studies exploring the connection between symptom relief and TME reprogramming will be essential to integrate acupuncture effectively into comprehensive cancer care.

### 5.2 The advantage of acupuncture in perioperative care and long-term survivorship

Breast cancer patients frequently experience multiple, interconnected symptoms across various phases of treatment. These symptoms can act synergistically, thereby significantly impairing both quality of life and long-term outcomes. Research suggests that acupuncture exerts multifaceted advantages in integrated breast cancer management, mediated through neuromodulatory and anti-inflammatory actions from acupoints to target organs. During the perioperative period, acupuncture has been shown to not only reduce the incidence of chronic pain at six months post-mastectomy but also effectively decrease arm circumference and the sensation of swelling in lymphedema patients (124, 125). Furthermore, survivors who have completed comprehensive treatment often experience psychoneurological symptoms and persistent treatment-related side effects. These include sequelae of systemic therapy, fatigue, breast symptoms, sleep disturbances, and arm morbidity. Consequently, acupuncture is commonly utilized to manage this diverse symptom cluster in long-term survivorship care. A cross-sectional survey of 415 breast cancer survivors revealed that 82.1% of participants reported symptom improvement following acupuncture intervention (126). Collectively, these findings underscore the feasibility and potential of acupuncture within integrative survivorship care for symptom management, highlighting its role in improving the long-term prognosis of breast cancer patients.

## 6 Discussion

Acupuncture has achieved worldwide clinical acceptance because it is simple, safe and demonstrably effective. In oncology, trials have concentrated on symptomatic indications—pain, fatigue, nausea, hot flushes, neuropathy, myelosuppression and insomnia—and have employed manual, electro-, auricular or transcutaneous stimulation as well as moxibustion. Although an expanding corpus of randomized controlled trials supports its utility, robust evidence remains restricted to a narrow range of indications. Methodological challenges are intrinsic to acupuncture research: intervention standardization, sham control credibility, outcome selection and statistical powering all remain contested. Integrative studies employing molecular biology, single-cell cytomics and genomics now indicate that acupuncture can do more than palliate; it can directly restrain tumor growth by reprogramming the tumor micro-environment—augmenting apoptosis, quelling inflammation, activating immune cells, normalizing vasculature and improving drug delivery—effects that appear to be orchestrated through neuro-immune crosstalk. Yet mechanistic inquiry remains fragmentary, with most reports confined to immune modulation and few interrogating proximal signaling cascades or systems-level networks. Pre-clinical data sets are heterogeneous and under-powered, precluding rigorous dose–response definitions or immediate translational guidance.

The tumor microenvironment (TME) governs initiation, progression and therapeutic response, rendering it a prime anti-cancer target (127). Here we systematically dissect how acupuncture reshapes the TME to restrain malignancy. Acupuncture both triggers tumor-cell apoptosis and synergizes with cytotoxics while concurrently reprogramming the tumor-immune microenvironment (TIME) through multifarious pathways. These encompass amplifying T-cell and NK-cell infiltration, enforcing M1 macrophage polarization, bolstering dendritic-cell competence and tuning B-cell and mast-cell activity. Moreover, acupuncture normalizes tumor vasculature by reprogramming endothelial cells and pericytes, thereby augmenting drug delivery and anti-tumor efficacy. Collectively, these actions constitute a multi-scale network that underpins acupuncture-mediated TME reprogramming. For the surgical oncologist, these mechanisms translate into tangible clinical opportunities. The ability of acupuncture to manage perioperative symptoms can contribute to improved quality of life and potentially enhance long-term survival. Its efficacy in reducing postoperative pain and addressing survivorship issues such as lymphedema aligns with the growing emphasis on patient-centered, functional outcomes in breast cancer care. Therefore, future clinical trials should not only focus on traditional survival endpoints but also incorporate robust metrics of symptom management, functional recovery, and patient-reported outcomes, particularly in perioperative and survivorship settings.

Nevertheless, pivotal challenges persist. Dose–response curves remain undefined and standardized end-point frameworks are absent. Mechanistic enquiries have privileged immunomodulation, leaving proximal signaling cascades and neuro-endocrine-immune synapses largely uncharted. I Moreover, bench-to-bedside translation is sparse and rigorously powered randomized controlled trials remain scarce. Future work must fuse systems biology, immunology and neuroscience to decode the molecular and cellular circuitry through which acupuncture rewires the TME. Concurrently, standardized yet individualized protocols should be forged and validated in adequately powered trials that delineate synergies with cytotoxics, immune checkpoint inhibitors and other modalities across molecularly stratified cohorts. Collectively, acupuncture stands ready to assume a precise and impactful role within tomorrow’s multimodal oncology armamentarium.

## References

[1] Siegel RL Kratzer TB Giaquinto AN Sung H Jemal A . Cancer statistics, 2025. CA Cancer J Clin. (2025) 75:10–45. doi: 10.3322/caac.21871, PMID: 39817679 PMC11745215

[2] Lu Z Chen Y Liu D Jiao X Liu C Wang Y . The landscape of cancer research and cancer care in China. Nat Med. (2023) 29:3022–32. doi: 10.1038/s41591-023-02655-3, PMID: 38087112

[3] Wahida A Buschhorn L Fröhling S Jost PJ Schneeweiss A Lichter P . The coming decade in precision oncology: six riddles. Nat Rev Cancer. (2023) 23:43–54. doi: 10.1038/s41568-022-00529-3, PMID: 36434139

[4] Zhou F Guo H Xia Y Le X Tan DSW Ramalingam SS . The changing treatment landscape of EGFR-mutant non-small-cell lung cancer. Nat Rev Clin Oncol. (2025) 22:95–116. doi: 10.1038/s41571-024-00971-2, PMID: 39614090

[5] Swanton C Bernard E Abbosh C André F Auwerx J Balmain A . Embracing cancer complexity: Hallmarks of systemic disease. Cell. (2024) 187:1589–616. doi: 10.1016/j.cell.2024.02.009, PMID: 38552609 PMC12077170

[6] Xiao Y Yu D . Tumor microenvironment as a therapeutic target in cancer. Pharmacol Ther. (2021) 221:107753. doi: 10.1016/j.pharmthera.2020.107753, PMID: 33259885 PMC8084948

[7] Pitt JM Marabelle A Eggermont A Soria JC Kroemer G Zitvogel L . Targeting the tumor microenvironment: removing obstruction to anticancer immune responses and immunotherapy. Ann Oncol. (2016) 27:1482–92. doi: 10.1093/annonc/mdw168, PMID: 27069014

[8] Das SS Bharadwaj P Bilal M Barani M Rahdar A Taboada P . Stimuli-responsive polymeric nanocarriers for drug delivery, imaging, and theragnosis. Polym (Basel). (2020) 12:1397. doi: 10.3390/polym12061397, PMID: 32580366 PMC7362228

[9] Liu J Dong S Liu S . Aberrant parasympathetic responses in acupuncture therapy for restoring immune homeostasis. Acupunct Herbal Med. (2023) 3:69–75.

[10] Li N Guo Y Gong Y Zhang Y Fan W Yao K . The anti-inflammatory actions and mechanisms of acupuncture from acupoint to target organs via neuro-immune regulation. J Inflammation Res. (2021) 14:7191–224. doi: 10.2147/jir.S341581, PMID: 34992414 PMC8710088

[11] Garcia MK McQuade J Haddad R Patel S Lee R Yang P . Systematic review of acupuncture in cancer care: a synthesis of the evidence. J Clin Oncol. (2013) 31:952–60. doi: 10.1200/jco.2012.43.5818, PMID: 23341529 PMC3577953

[12] Li S Zhao S Guo Y Yang Y Huang J Wang J . Clinical efficacy and potential mechanisms of acupoint stimulation combined with chemotherapy in combating cancer: A review and prospects. Front Oncol. (2022) 12:864046. doi: 10.3389/fonc.2022.864046, PMID: 35547876 PMC9082419

[13] Li YW Li W Wang ST Gong YN Dou BM Lyu ZX . The autonomic nervous system: A potential link to the efficacy of acupuncture. Front Neurosci. (2022) 16:1038945. doi: 10.3389/fnins.2022.1038945, PMID: 36570846 PMC9772996

[14] Wang N Zhao L Zhang D Kong F . Research progress on the immunomodulatory mechanism of acupuncture in tumor immune microenvironment. Front Immunol. (2023) 14:1092402. doi: 10.3389/fimmu.2023.1092402, PMID: 36865562 PMC9971227

[15] Hanahan D Weinberg RA . The hallmarks of cancer. Cell. (2000) 100:57–70. doi: 10.1016/s0092-8674(00)81683-9, PMID: 10647931

[16] Alese OB Zhou W Jiang R Zakka K Huang Z Okoli C . Predictive and prognostic effects of primary tumor size on colorectal cancer survival. Front Oncol. (2021) 11:728076. doi: 10.3389/fonc.2021.728076, PMID: 34956863 PMC8695445

[17] Shi X Wang X Yao W Shi D Shao X Lu Z . Mechanism insights and therapeutic intervention of tumor metastasis: latest developments and perspectives. Signal Transduct Target Ther. (2024) 9:192. doi: 10.1038/s41392-024-01885-2, PMID: 39090094 PMC11294630

[18] Lai M Wang SM Zhang WL Wang Y Huang SQ Dong W . Effects of electroacupuncture on tumor growth and immune function in the Walker-256 model rat. Zhongguo Zhen Jiu. (2008) 28:607–9. 18767588

[19] Xu X Feng X He M Zhang Z Wang J Zhu H . The effect of acupuncture on tumor growth and gut microbiota in mice inoculated with osteosarcoma cells. Chin Med. (2020) 15:33. doi: 10.1186/s13020-020-00315-z, PMID: 32292489 PMC7140491

[20] Smeester BA Al-Gizawiy M O’Brien EE Ericson ME Triemstra JL Beitz AJ . The effect of electroacupuncture on osteosarcoma tumor growth and metastasis: analysis of different treatment regimens. Evid Based Complement Alternat Med. (2013) 2013:387169. doi: 10.1155/2013/387169, PMID: 24228059 PMC3818845

[21] Gotwals P Cameron S Cipolletta D Cremasco V Crystal A Hewes B . Prospects for combining targeted and conventional cancer therapy with immunotherapy. Nat Rev Cancer. (2017) 17:286–301. doi: 10.1038/nrc.2017.17, PMID: 28338065

[22] Saraswati W Dahlan EG Saputra K Sutrisno TC . Effect of electroacupuncture on natural-killer cells and tumor size in patients with cervical squamous-cell carcinoma: A randomized controlled trial. Med Acupunct. (2019) 31:29–36. doi: 10.1089/acu.2018.1316, PMID: 30805077 PMC6386775

[23] Li ZL Zeng WJ Li GH Zhang JT Li ZC Wang SY . Effect of electroacupuncture of “Zusanli” (ST36) combined with capeOX on apoptosis and ferroptosis in nude mice with colorectal cancer. Zhen Ci Yan Jiu. (2024) 49:678–85. doi: 10.13702/j.1000-0607.20230990, PMID: 39020485

[24] Li S Chen X Shi H Yi M Xiong B Li T . Tailoring traditional Chinese medicine in cancer therapy. Mol Cancer. (2025) 24:27. doi: 10.1186/s12943-024-02213-6, PMID: 39838407 PMC11749133

[25] Ma K Wang L Li W Tang T Ma B Zhang L . Turning cold into hot: emerging strategies to fire up the tumor microenvironment. Trends Cancer. (2025) 11:117–34. doi: 10.1016/j.trecan.2024.11.011, PMID: 39730243

[26] Camus M Tosolini M Mlecnik B Pagès F Kirilovsky A Berger A . Coordination of intratumoral immune reaction and human colorectal cancer recurrence. Cancer Res. (2009) 69:2685–93. doi: 10.1158/0008-5472.Can-08-2654, PMID: 19258510

[27] Szentkereszty M Ladányi A Gálffy G Tóvári J Losonczy G . Density of tumor-infiltrating NK and Treg cells is associated with 5 years progression-free and overall survival in resected lung adenocarcinoma. Lung Cancer. (2024) 192:107824. doi: 10.1016/j.lungcan.2024.107824, PMID: 38761665

[28] Liu J He X Deng S Zhao S Zhang S Chen Z . QDPR deficiency drives immune suppression in pancreatic cancer. Cell Metab. (2024) 36:984–999.e8. doi: 10.1016/j.cmet.2024.03.015, PMID: 38642552

[29] Wang M Liu W Ge J Liu S . The immunomodulatory mechanisms for acupuncture practice. Front Immunol. (2023) 14:1147718. doi: 10.3389/fimmu.2023.1147718, PMID: 37090714 PMC10117649

[30] Chi H Pepper M Thomas PG . Principles and therapeutic applications of adaptive immunity. Cell. (2024) 187:2052–78. doi: 10.1016/j.cell.2024.03.037, PMID: 38670065 PMC11177542

[31] Zebley CC Zehn D Gottschalk S Chi H . T cell dysfunction and therapeutic intervention in cancer. Nat Immunol. (2024) 25:1344–54. doi: 10.1038/s41590-024-01896-9, PMID: 39025962 PMC11616736

[32] Gomez-Brouchet A Illac C Gilhodes J Bouvier C Aubert S Guinebretiere JM . CD163-positive tumor-associated macrophages and CD8-positive cytotoxic lymphocytes are powerful diagnostic markers for the therapeutic stratification of osteosarcoma patients: An immunohistochemical analysis of the biopsies fromthe French OS2006 phase 3 trial. Oncoimmunology. (2017) 6:e1331193. doi: 10.1080/2162402x.2017.1331193, PMID: 28932633 PMC5599091

[33] Donizy P Wu CL Kopczynski J Pieniazek M Biecek P Ryś J . Prognostic role of tumoral PD-L1 and IDO1 expression, and intratumoral CD8+ and foxP3+ Lymphocyte infiltrates in 132 primary cutaneous merkel cell carcinomas. Int J Mol Sci. (2021) 22. doi: 10.3390/ijms22115489, PMID: 34071045 PMC8197111

[34] McLane LM Abdel-Hakeem MS Wherry EJ . CD8 T cell exhaustion during chronic viral infection and cancer. Annu Rev Immunol. (2019) 37:457–95. doi: 10.1146/annurev-immunol-041015-055318, PMID: 30676822

[35] Edwards J Wilmott JS Madore J Gide TN Quek C Tasker A . CD103(+) tumor-resident CD8(+) T cells are associated with improved survival in immunotherapy-naïve melanoma patients and expand significantly during anti-PD-1 treatment. Clin Cancer Res. (2018) 24:3036–45. doi: 10.1158/1078-0432.Ccr-17-2257, PMID: 29599411

[36] Montauti E Oh DY Fong L . CD4(+) T cells in antitumor immunity. Trends Cancer. (2024) 10:969–85. doi: 10.1016/j.trecan.2024.07.009, PMID: 39242276 PMC11464182

[37] Abdeladhim M Karnell JL Rieder SA . In or out of control: Modulating regulatory T cell homeostasis and function with immune checkpoint pathways. Front Immunol. (2022) 13:1033705. doi: 10.3389/fimmu.2022.1033705, PMID: 36591244 PMC9799097

[38] Liang Y Du JY Fang JF Fang RY Zhou J Shao XM . Alleviating mechanical allodynia and modulating cellular immunity contribute to electroacupuncture’s dual effect on bone cancer pain. Integr Cancer Ther. (2018) 17:401–10. doi: 10.1177/1534735417728335, PMID: 28870114 PMC6041932

[39] Tai JB Hong L Ma ME Xu J Fang JQ Jiang YQ . Evaluation of therapeutic effect of transcutaneous electrical acupoint stimulation on bone metastasis pain and its influence on immune function of patients. Ann Palliat Med. (2020) 9:2538–44. doi: 10.21037/apm-19-434, PMID: 32954744

[40] Ye F Chen S Liu W . Effects of electro-acupuncture on immune function after chemotherapy in 28 cases. J Tradit Chin Med. (2002) 22:21–3. 11977512

[41] Wang J Yang Y Lu S Huang J Li S Chang H . Electroacupuncture combined with cisplatin induces an effective anti-tumor immune response by protecting chemotherapy-impaired bone marrow hematopoiesis in non–small cell lung cancer mice. Acupunct Herbal Med. (2025) 5:229–45.

[42] Li S Huang J Guo Y Wang J Lu S Wang B . PAC1 receptor mediates electroacupuncture-induced neuro and immune protection during cisplatin chemotherapy. Front Immunol. (2021) 12:714244. doi: 10.3389/fimmu.2021.714244, PMID: 34552585 PMC8450570

[43] Agarwal S Garg R Minhas V Bhatnagar S Mishra S Kumar V . To assess the Prevalence and Predictors of Cancer-related Fatigue and its Impact on Quality of Life in Advanced Cancer Patients Receiving Palliative Care in a Tertiary Care Hospital: A Cross-sectional Descriptive Study. Indian J Palliat Care. (2020) 26:523–7. doi: 10.4103/ijpc.Ijpc_223_19, PMID: 33623316 PMC7888426

[44] Pedraz-Petrozzi B Neumann E Sammer G . Pro-inflammatory markers and fatigue in patients with depression: A case-control study. Sci Rep. (2020) 10:9494. doi: 10.1038/s41598-020-66532-6, PMID: 32528052 PMC7289841

[45] Li J Fu R Guo X Pan Z Xie J . Acupuncture improves immunity and fatigue after chemotherapy in breast cancer patients by inhibiting the Leptin/AMPK signaling pathway. Support Care Cancer. (2023) 31:506. doi: 10.1007/s00520-023-07967-1, PMID: 37542585 PMC10404187

[46] Toh YL Tan CJ Yeo AHL Shwe M Ho HK Gan YX . Association of plasma leptin, pro-inflammatory adipokines and cancer-related fatigue in early-stage breast cancer patients: A prospective cohort study. J Cell Mol Med. (2019) 23:4281–9. doi: 10.1111/jcmm.14319, PMID: 31016867 PMC6533466

[47] Tian Y Ma Y Li X Lu G Wang S Qiu X . Electroacupuncture combined with HDAC1 inhibitor suppress tumor growth via improving the recruitment of intratumor CD8(+) T cells for triple-negative breast cancer in mice. Front Oncol. (2025) 15:1584722. doi: 10.3389/fonc.2025.1584722, PMID: 40475010 PMC12137248

[48] Fan Y Ji X Yuan K Wu Q Lou M . HDAC1 mediates immunosuppression of the tumor microenvironment in non-small cell lung cancer. J Inflammation Res. (2025) 18:3333–47. doi: 10.2147/jir.S509316, PMID: 40078575 PMC11900795

[49] Ganesh K Stadler ZK Cercek A Mendelsohn RB Shia J Segal NH . Immunotherapy in colorectal cancer: rationale, challenges and potential. Nat Rev Gastroenterol Hepatol. (2019) 16:361–75. doi: 10.1038/s41575-019-0126-x, PMID: 30886395 PMC7295073

[50] Wang Y Liu F Du X Shi J Yu R Li S . Combination of anti-PD-1 and electroacupuncture induces a potent antitumor immune response in microsatellite-stable colorectal cancer. Cancer Immunol Res. (2024) 12:26–35. doi: 10.1158/2326-6066.Cir-23-0309, PMID: 37956404

[51] Liu S Wang ZF Su YS Ray RS Jing XH Wang YQ . Somatotopic organization and intensity dependence in driving distinct NPY-expressing sympathetic pathways by electroacupuncture. Neuron. (2020) 108:436–450.e7. doi: 10.1016/j.neuron.2020.07.015, PMID: 32791039 PMC7666081

[52] Shi X Cheng X Jiang A Shi W Zhu L Mou W . Immune checkpoints in B cells: unlocking new potentials in cancer treatment. Adv Sci (Weinh). (2024) 11:e2403423. doi: 10.1002/advs.202403423, PMID: 39509319 PMC11653663

[53] Ye F Liu D Wang S Xu L . Effects of electro-acupuncture on T cell subpopulations, NK activity, humoral immunity and leukocyte count in patients undergoing chemotherapy. J Tradit Chin Med. (2007) 27:19–21. 17393618

[54] Cabioglu MT Ergene N Surucu HS Celik HH Findik D . Serum IgG, IgA, IgM, and IgE levels after electroacupuncture and diet therapy in obese women. Am J Chin Med. (2007) 35:955–65. doi: 10.1142/s0192415x07005429, PMID: 18186582

[55] Yim YK Lee H Hong KE Kim YI Lee BR Son CG . Electro-acupuncture at acupoint ST36 reduces inflammation and regulates immune activity in Collagen-Induced Arthritic Mice. Evid Based Complement Alternat Med. (2007) 4:51–7. doi: 10.1093/ecam/nel054, PMID: 17342241 PMC1810363

[56] Liu W Zhong B Wagner RW Garcia MK McQuade JL Huang W . Systematic review and meta-analysis of acupuncture for modulation of immune and inflammatory markers in cancer patients. Integr Cancer Ther. (2024) 23:15347354241302072. doi: 10.1177/15347354241302072, PMID: 39663880 PMC11635873

[57] Carpenter S O’Neill LAJ . From periphery to center stage: 50 years of advancements in innate immunity. Cell. (2024) 187:2030–51. doi: 10.1016/j.cell.2024.03.036, PMID: 38670064 PMC11060700

[58] Warrick KA Vallez CN Meibers HE Pasare C . Bidirectional communication between the innate and adaptive immune systems. Annu Rev Immunol. (2025) 43:489–514. doi: 10.1146/annurev-immunol-083122-040624, PMID: 40279312 PMC12120936

[59] Li C Yu X Han X Lian C Wang Z Shao S . Innate immune cells in tumor microenvironment: A new frontier in cancer immunotherapy. iScience. (2024) 27:110750. doi: 10.1016/j.isci.2024.110750, PMID: 39280627 PMC11399700

[60] Vivier E Rebuffet L Narni-Mancinelli E Cornen S Igarashi RY Fantin VR . Natural killer cell therapies. Nature. (8000) 2024:626. doi: 10.1038/s41586-023-06945-1, PMID: 38383621

[61] Morcillo-Martín-Romo P Valverde-Pozo J Ortiz-Bueno M Arnone M Espinar-Barranco L Espinar-Barranco C . The role of NK cells in cancer immunotherapy: mechanisms, evasion strategies, and therapeutic advances. Biomedicines. (2025) 13:857. doi: 10.3390/biomedicines13040857, PMID: 40299429 PMC12024875

[62] Neo SY Tong L Chong J Liu Y Jing X Oliveira MMS . Tum or-associated NK cells drive MDSC-mediated tumor immune tolerance through the IL-6/STAT3 axis. Sci Transl Med. (2024) 16:eadi2952. doi: 10.1126/scitranslmed.adi2952, PMID: 38748775

[63] Arranz L Guayerbas N Siboni L de la Fuente M . Effect of acupuncture treatment on the immune function impairment found in anxious women. Am J Chin Med. (2007) 35:35–51. doi: 10.1142/s0192415x07004606, PMID: 17265549

[64] Li G Li S An L Wang B . Electroacupuncture alleviates intraoperative immunosuppression in patients undergoing supratentorial craniotomy. Acupunct Med. (2013) 31:51–6. doi: 10.1136/acupmed-2012-010254, PMID: 23315447

[65] Johnston MF Ortiz Sánchez E Vujanovic NL Li W . Acupuncture may stimulate anticancer immunity via activation of natural killer cells. Evid Based Complement Alternat Med. (2011) 2011:481625. doi: 10.1093/ecam/nep236, PMID: 21785626 PMC3135660

[66] Zhang SY Du YQ . Effects of warming needle moxibustion on improvement of gastrointestinal and immune function in patients with postoperation of colorectal cancer. Zhongguo Zhen Jiu. (2011) 31:513–7. 21739693

[67] Tu Q Yang Z Gan J Zhang J Que B Song Q . Transcutaneous electrical acupoint stimulation improves immunological function during the perioperative period in patients with non-small cell lung cancer undergoing video-assisted thoracic surgical lobectomy. Technol Cancer Res Treat. (2018) 17:1533033818806477. doi: 10.1177/1533033818806477, PMID: 30381011 PMC6259054

[68] Huang Z Hu Z Ouyang J Huang C . Electroacupuncture regulates the DREAM/NF-κB signalling pathway and ameliorates cyclophosphamide-induced immunosuppression in mice. Acupunct Med. (2019) 37:292–300. doi: 10.1136/acupmed-2017-011593, PMID: 31192694

[69] Liu F Wang Y Lyu K Du X Zhou M Shi J . Acupuncture and its ability to restore and maintain immune homeostasis. Qjm. (2024) 117:167–76. doi: 10.1093/qjmed/hcad134, PMID: 37318994

[70] Cho ZH Hwang SC Wong EK Son YD Kang CK Park TS . Neural substrates, experimental evidences and functional hypothesis of acupuncture mechanisms. Acta Neurol Scand. (2006) 113:370–7. doi: 10.1111/j.1600-0404.2006.00600.x, PMID: 16674603

[71] Dokur M Chen CP Advis JP Sarkar DK . Beta-endorphin modulation of interferon-gamma, perforin and granzyme B levels in splenic NK cells: effects of ethanol. J Neuroimmunol. (2005) 166:29–38. doi: 10.1016/j.jneuroim.2005.03.015, PMID: 16005984

[72] Yu Y Kasahara T Sato T Asano K Yu G Fang J . Role of endogenous interferon-gamma on the enhancement of splenic NK cell activity by electroacupuncture stimulation in mice. J Neuroimmunol. (1998) 90:176–86. doi: 10.1016/s0165-5728(98)00143-x, PMID: 9817445

[73] Zhang Z Yu Q Zhang X Wang X Su Y He W . Electroacupuncture regulates inflammatory cytokines by activating the vagus nerve to enhance antitumor immunity in mice with breast tumors. Life Sci. (2021) 272:119259. doi: 10.1016/j.lfs.2021.119259, PMID: 33636172

[74] Wong NR Mohan J Kopecky BJ Guo S Du L Leid J . Resident cardiac macrophages mediate adaptive myocardial remodeling. Immunity. (2021) 54:2072–2088.e7. doi: 10.1016/j.immuni.2021.07.003, PMID: 34320366 PMC8446343

[75] Han C Deng Y Xu W Liu Z Wang T Wang S . The roles of tumor-associated macrophages in prostate cancer. J Oncol. (2022) 2022:8580043. doi: 10.1155/2022/8580043, PMID: 36117852 PMC9473905

[76] Yang Y Li S To KKW Zhu S Wang F Fu L . Tumor-associated macrophages remodel the suppressive tumor immune microenvironment and targeted therapy for immunotherapy. J Exp Clin Cancer Res. (2025) 44:145. doi: 10.1186/s13046-025-03377-9, PMID: 40380196 PMC12083052

[77] Hinshaw DC Hanna A Lama-Sherpa T Metge B Kammerud SC Benavides GA . Hedgehog signaling regulates metabolism and polarization of mammary tumor-associated macrophages. Cancer Res. (2021) 81:5425–37. doi: 10.1158/0008-5472.Can-20-1723, PMID: 34289986 PMC8563376

[78] Zhang XF Xiang SY Lu J Li Y Zhao SJ Jiang CW . Electroacupuncture inhibits IL-17/IL-17R and post-receptor MAPK signaling pathways in a rat model of chronic obstructive pulmonary disease. Acupunct Med. (2021) 39:663–72. doi: 10.1177/0964528421996720, PMID: 33715422

[79] Yang F Gong Y Yu N Yao L Zhao X Hong S . ST36 acupuncture alleviates the inflammation of adjuvant-induced arthritic rats by targeting monocyte/macrophage modulation. Evid Based Complement Alternat Med. (2021) 2021:9430501. doi: 10.1155/2021/9430501, PMID: 33727948 PMC7936911

[80] Qi X Lian Y Fan Z Wang H Jiang H He M . Electroacupuncture normalized tumor vasculature by downregulating glyoxalase-1 to polarize tumor-associated macrophage to M1 phenotype in triple-negative breast cancer. Int Immunopharmacol. (2025) 147:113988. doi: 10.1016/j.intimp.2024.113988, PMID: 39778275

[81] Wan YX Qi XW Lian YY Liu ZY Wang H Qiu YQ . Electroacupuncture facilitates vascular normalization by inhibiting Glyoxalase1 in endothelial cells to attenuate glycolysis and angiogenesis in triple-negative breast cancer. Cancer Lett. (2024) 598:217094. doi: 10.1016/j.canlet.2024.217094, PMID: 38945204

[82] Shen M Li Z Wang J Xiang H Xie Q . Traditional Chinese herbal medicine: harnessing dendritic cells for anti-tumor benefits. Front Immunol. (2024) 15:1408474. doi: 10.3389/fimmu.2024.1408474, PMID: 39364399 PMC11446781

[83] Xiao Z Wang J Yang J Guo F Zhang L Zhang L . Dendritic cells instruct T cell anti-tumor immunity and immunotherapy response. Innovation Med. (2025) 3:100128–1-100128-18.

[84] Xu X Wang N Li Y Fan S Mu B Zhu J . Acupuncture potentiates anti-PD-1 efficacy by promoting CD5(+) dendritic cells and T cell-mediated tumor immunity in a mouse model of breast cancer. Nutr Cancer. (2025) 16:1–14. doi: 10.1080/01635581.2025.2517737, PMID: 40521704

[85] He M Roussak K Ma F Borcherding N Garin V White M . CD5 expression by dendritic cells directs T cell immunity and sustains immunotherapy responses. Science. (2023) 379:eabg2752. doi: 10.1126/science.abg2752, PMID: 36795805 PMC10424698

[86] Dong H Zhong Z Chen W Wu X Zhang Q Huang G . Effect of acupuncture on endometrial angiogenesis and uterus dendritic cells in COH rats during peri-implantation period. Evid Based Complement Alternat Med. (2017) 2017:3647080. doi: 10.1155/2017/3647080, PMID: 28588637 PMC5446881

[87] Traina G . Mast cells in human health and diseases. Int J Mol Sci. (2023) 24:6668. doi: 10.3390/ijms24076668, PMID: 37047641 PMC10095135

[88] Guo X Sun M Yang P Meng X Liu R . Role of mast cells activation in the tumor immune microenvironment and immunotherapy of cancers. Eur J Pharmacol. (2023) 960:176103. doi: 10.1016/j.ejphar.2023.176103, PMID: 37852570

[89] Shu F Yu J Liu Y Wang F Gou G Wen M . Mast cells: key players in digestive system tumors and their interactions with immune cells. Cell Death Discov. (2025) 11:8. doi: 10.1038/s41420-024-02258-y, PMID: 39814702 PMC11735678

[90] Yu D Zhu J Zhu M Wei K Chen Q Wu X . Inhibition of mast cell degranulation relieves visceral hypersensitivity induced by pancreatic carcinoma in mice. J Mol Neurosci. (2019) 69:235–45. doi: 10.1007/s12031-019-01352-6, PMID: 31201657 PMC6732154

[91] Longo V Tamma R Brunetti O Pisconti S Argentiero A Silvestris N . Mast cells and angiogenesis in pancreatic ductal adenocarcinoma. Clin Exp Med. (2018) 18:319–23. doi: 10.1007/s10238-018-0493-6, PMID: 29492715

[92] Wu ML Xu DS Bai WZ Cui JJ Shu HM He W . Local cutaneous nerve terminal and mast cell responses to manual acupuncture in acupoint LI4 area of the rats. J Chem Neuroanat. (2015) 68:14–21. doi: 10.1016/j.jchemneu.2015.06.002, PMID: 26148746

[93] Wang Z Lu M Ren J Wu X Long M Chen L . Electroacupuncture inhibits mast cell degranulation via cannabinoid CB2 receptors in a rat model of allergic contact dermatitis. Acupunct Med. (2019) 37:348–55. doi: 10.1136/acupmed-2017-011506, PMID: 31429590

[94] Sun MZ Wang X Li YC Liu YH Yu Y Ren LJ . Effects of acupuncture needle modification on acupuncture analgesia. J Integr Med. (2025) 23:66–78. doi: 10.1016/j.joim.2024.11.007, PMID: 39675938

[95] Jiang X Wang J Deng X Xiong F Zhang S Gong Z . The role of microenvironment in tumor angiogenesis. J Exp Clin Cancer Res. (2020) 39:204. doi: 10.1186/s13046-020-01709-5, PMID: 32993787 PMC7526376

[96] Folkman J . Tumor angiogenesis: therapeutic implications. N Engl J Med. (1971) 285:1182–6. doi: 10.1056/nejm197111182852108, PMID: 4938153

[97] De Palma M Biziato D Petrova TV . Microenvironmental regulation of tumour angiogenesis. Nat Rev Cancer. (2017) 17:457–74. doi: 10.1038/nrc.2017.51, PMID: 28706266

[98] Lugano R Ramachandran M Dimberg A . Tumor angiogenesis: causes, consequences, challenges and opportunities. Cell Mol Life Sci. (2020) 77:1745–70. doi: 10.1007/s00018-019-03351-7, PMID: 31690961 PMC7190605

[99] Wei X Chen Y Jiang X Peng M Liu Y Mo Y . Mechanisms of vasculogenic mimicry in hypoxic tumor microenvironments. Mol Cancer. (2021) 20:7. doi: 10.1186/s12943-020-01288-1, PMID: 33397409 PMC7784348

[100] Cheng Y Li J Feng X Wu Y Wu X Lau BWM . Taohong Siwu decoction enhances the chemotherapeutic efficacy of doxorubicin by promoting tumor vascular normalization. Phytomedicine. (2024) 134:155995. doi: 10.1016/j.phymed.2024.155995, PMID: 39270591

[101] Jain RK . Normalizing tumor vasculature with anti-angiogenic therapy: a new paradigm for combination therapy. Nat Med. (2001) 7:987–9. doi: 10.1038/nm0901-987, PMID: 11533692

[102] Dudley AC . Tumor endothelial cells. Cold Spring Harb Perspect Med. (2012) 2:a006536. doi: 10.1101/cshperspect.a006536, PMID: 22393533 PMC3282494

[103] De Bock K Cauwenberghs S Carmeliet P . Vessel abnormalization: another hallmark of cancer? Molecular mechanisms and therapeutic implications. Curr Opin Genet Dev. (2011) 21:73–9. doi: 10.1016/j.gde.2010.10.008, PMID: 21106363

[104] Guelfi S Hodivala-Dilke K Bergers G . Targeting the tumour vasculature: from vessel destruction to promotion. Nat Rev Cancer. (2024) 24:655–75. doi: 10.1038/s41568-024-00736-0, PMID: 39210063

[105] Wilhelm K Happel K Eelen G Schoors S Oellerich MF Lim R . FOXO1 couples metabolic activity and growth state in the vascular endothelium. Nature. (2016) 529:216–20. doi: 10.1038/nature16498, PMID: 26735015 PMC5380221

[106] Cantelmo AR Conradi LC Brajic A Goveia J Kalucka J Pircher A . Inhibition of the glycolytic activator PFKFB3 in endothelium induces tumor vessel normalization, impairs metastasis, and improves chemotherapy. Cancer Cell. (2016) 30:968–85. doi: 10.1016/j.ccell.2016.10.006, PMID: 27866851 PMC5675554

[107] Goel HL Mercurio AM . VEGF targets the tumour cell. Nat Rev Cancer. (2013) 13:871–82. doi: 10.1038/nrc3627, PMID: 24263190 PMC4011842

[108] Jiang X Tian Y Xu L Zhang Q Wan Y Qi X . Inhibition of triple-negative breast cancer tumor growth by electroacupuncture with encircled needling and its mechanisms in a mice xenograft model. Int J Med Sci. (2019) 16:1642–51. doi: 10.7150/ijms.38521, PMID: 31839752 PMC6909807

[109] Raimondi C . Neuropilin-1 enforces extracellular matrix signalling via ABL1 to promote angiogenesis. Biochem Soc Trans. (2014) 42:1429–34. doi: 10.1042/bst20140141, PMID: 25233427

[110] Tan AC Ashley DM López GY Malinzak M Friedman HS Khasraw M . Management of glioblastoma: State of the art and future directions. CA Cancer J Clin. (2020) 70:299–312. doi: 10.3322/caac.21613, PMID: 32478924

[111] Gong Z Zhou D Wu D Han Y Yu H Shen H . Challenges and material innovations in drug delivery to central nervous system tumors. Biomaterials. (2025) 319:123180. doi: 10.1016/j.biomaterials.2025.123180, PMID: 39985979

[112] Ma C Ye Q Qian K Dai M Gan L Yang J . Anti-glioma effect of paclitaxel mediated by specific mode electroacupuncture stimulation and the related role of the Hedgehog pathway. Brain Res Bull. (2024) 213:110985. doi: 10.1016/j.brainresbull.2024.110985, PMID: 38806118

[113] Gong P Zhang S Ren L Zhang J Zhao Y Mao X . Electroacupuncture of the trigeminal nerve causes N-methyl-D-aspartate receptors to mediate blood-brain barrier opening and induces neuronal excitatory changes. Front Cell Neurosci. (2022) 16:1020644. doi: 10.3389/fncel.2022.1020644, PMID: 36313622 PMC9606778

[114] Soh KS . Hypothesis on the treatment of gliomas with acupuncture at the primo node corresponding to zusanli (ST 36). Med Acupunct. (2015) 27:144–50. doi: 10.1089/acu.2014.1089, PMID: 26155319 PMC4491168

[115] Zhang C Du Z Gao Y Lim KS Zhou W Huang H . Methionine secreted by tumor-associated pericytes supports cancer stem cells in clear cell renal carcinoma. Cell Metab. (2024) 36:778–792.e10. doi: 10.1016/j.cmet.2024.01.018, PMID: 38378000

[116] Meng MB Zaorsky NG Deng L Wang HH Chao J Zhao LJ . Pericytes: a double-edged sword in cancer therapy. Future Oncol. (2015) 11:169–79. doi: 10.2217/fon.14.123, PMID: 25143028

[117] Xian X Håkansson J Ståhlberg A Lindblom P Betsholtz C Gerhardt H . Pericytes limit tumor cell metastasis. J Clin Invest. (2006) 116:642–51. doi: 10.1172/jci25705, PMID: 16470244 PMC1361347

[118] Yang M Wan Y Jiang X Qi X Wang L Liu Z . Electro-acupuncture promotes accumulation of paclitaxel by altering tumor microvasculature and microenvironment in breast cancer of mice. Front Oncol. (2019) 9:576. doi: 10.3389/fonc.2019.00576, PMID: 31312613 PMC6614178

[119] Zhang ZX Cheng ZD Li CR Ke AJ Chen JL Chen YG . Effects of acupuncture on distribution taxis of paclitaxel in mice with lung cancer. Zhongguo Zhen Jiu. (2014) 34:1208–13. 25876355

[120] Harris MA Savas P Virassamy B O'Malley MMR Kay J Mueller SN . Towards targeting the breast cancer immune microenvironment. Nat Rev Cancer. (2024) 24:554–77. doi: 10.1038/s41568-024-00714-6, PMID: 38969810

[121] Chen L Li H Zhang H Yang H Qian J Li Z . Camrelizumab vs placebo in combination with chemotherapy as neoadjuvant treatment in patients with early or locally advanced triple-negative breast cancer: the camRelief randomized clinical trial. Jama. (2025) 333:673–81. doi: 10.1001/jama.2024.23560, PMID: 39671272 PMC11862970

[122] Zhao H Tian ZZ Chen BY . Electroacupuncture stimulates hypothalamic aromatization. Brain Res. (2005) 1037:164–70. doi: 10.1016/j.brainres.2005.01.004, PMID: 15777765

[123] Zhao YX Yao MJ Liu Q Xin JJ Gao JH Yu XC . Electroacupuncture treatment attenuates paclitaxel-induced neuropathic pain in rats via inhibiting spinal glia and the TLR4/NF-κB pathway. J Pain Res. (2020) 13:239–50. doi: 10.2147/jpr.S241101, PMID: 32099448 PMC7005725

[124] Lu Z Wang Q Sun X Zhang W Min S Zhang J . Transcutaneous electrical acupoint stimulation before surgery reduces chronic pain after mastectomy: A randomized clinical trial. J Clin Anesth. (2021) 74:110453. doi: 10.1016/j.jclinane.2021.110453, PMID: 34271271

[125] Lu C Li GL Deng DH Bao WL Wang Y Zhang AQ . Transcutaneous electrical acupoint stimulation combined with warm acupuncture for breast cancer related upper limb lymphedema: A retrospective cohort study. Chin J Integr Med. (2023) 29:534–9. doi: 10.1007/s11655-022-3684-7, PMID: 36374440

[126] Zayas J Ruddy KJ Olson JE Couch FJ Bauer BA Mallory MJ . Real-world experiences with acupuncture among breast cancer survivors: a cross-sectional survey study. Support Care Cancer. (2020) 28:5833–8. doi: 10.1007/s00520-020-05442-9, PMID: 32253604 PMC7541443

[127] de Visser KE Joyce JA . The evolving tumor microenvironment: From cancer initiation to metastatic outgrowth. Cancer Cell. (2023) 41:374–403. doi: 10.1016/j.ccell.2023.02.016, PMID: 36917948

